# Periscope

**Published:** 1881-04

**Authors:** 


### PERISCOPE.

*The Homœopathic Times*, Jan. and Feb., 1881.

Dr. E. R. Corson writes upon the recent epidemic of Dengue in Savannah, and gives cases treated with *Acon.*, *Ars.*, *Bell.*, *Gels.*, *Nux v.*, *Hellebore*, *China*, *Ipecac.*, *Merc.-sol.*, *Sulph. ac.* and hot *Pediluvia.* The specific indications in each case are not given; only a few obscure generalities are collected at the end of the article. Morover, the remedies were sometimes given in alternation; hence no lessons of any clinical value can be learned from this essay.

Dr. W. A. Dewy, house physician at Ward's Island reports a case of traumatic erysipelas of the forearm complicated with mania-a-potu, acute desquamative nephritis and acute bronchitis. The patient on admission is semi-delirious; starts suddenly at any noise, jar or touch. Swelling of the arm is bright red. Dozing disturbed with startings and crying out; sleepy but can not sleep; dilated pupils; flushed face; throbbing in arm with soreness; body hot to the touch and on uncovering heat seems to steam out; feet cold, head hot. *Bell.* 3. The next day patient was seized with violent delirium and then the remedy was changed to *Hyos.* 3d. We think the doctor made a mistake in changing his prescription. He should have continued the *Bell.* and, possibly, have raised its potency. Afterward for the inflammation of the arm the patient received *Silic.* 30, then *Hepar.* 3. This last was repeated when the nephritis set in, until with the appearance of tube casts in the urine, œdema of legs, hurried breathing, large mucous rales all over the chest, and profuse expectoration of frothy mucus, he was given *Ant-tart.* 30. He got better under this remedy; then pain in right chest on deep breathing followed. *Bry.* 3 was now given and he now improved so rapidly that he was discharged, one month after his entrance to the hospital, cured.

Another man, addicted to excessive drinking and tainted with gonorrhœa and syphilis, was admitted, with erysipelas of the leg in the region of an old ulcer. *Bell.* 3 was given and the next day delirium occurred, whilst the inflammation showed improvement. Remedy changed to *Hyos.* He slowly improved under it and when the erysipelas had disappeared, was removed to the surgical wards for treatment of the ulcer. This was a mistake; the ulcer should have continued to receive drug treatment without any mechanical interference.

A third patient had traumatic erysipelas of scrotum, cured in two weeks with *Bell.* 3. An instructive case.

A fourth case cited was traumatic erysipelas of scalp from a wound. Cured with *Rhus t.* 3.

The fifth case was traumatic erysipelas of forearm from the bite of another man. Cured with *Bell.* 3.

Dr. Elias C. Price reports the cure of two cases of bursitis, or house-maids knee, with *Sticta-Pul.* He gives no indications, hence it is inferred that he proposes to use it in *every* case.

From *L'Art Medical*, for Aug., 1880, is translated an article by Dr. Jousset, reporting five cases of endocarditis, either cured or very much benefitted by infusion of *Digitalis*, after being unsuccessfully treated with other remedies singly and in alternation—not forgetting also the administration of *Morphia* by hypodermic injection. The treatment is empirical entirely.

Dr. H. C. Frost reports the case of a laborer who fell down a well upon the point of a stick, which passed through the scrotum, upward, between the abdominal muscles and adipose tissue, thence, between the muscles of the chest and the adipose tissue as far as the third rib where it emerged. He recovered in twenty-seven days.

Also successful removal of a fibro cartilaginous tumor from parotid region.

*United States Medical Investigator*, Jan 1st. and 15th.; Feb. 1st.

Dr. J. S. Mitchell writes upon the value of local applications of kerosene in phthisis, illustrated with cases. His method of prescribing is empirical. Hence there is no more to be learned from it than from a similar article in a journal of the old school. Having had success with *Petrol.* in the crude way he proposes to investigate its value when given in the higher dilutions. The doctor forgets that merely giving drugs in dilution is not necessarily homœopathic prescribing. The drug must be given in accordance with the law.

Dr. Q. O. Sutherland was called to a child accidentally poisoned with atropine. He saved the little sufferer by the exhibition of large doses of the "physiological antagonist" of the poison, *Sulphate of Morphia.* The doctor is aware that his "method of procedure is open to severe criticism, but he also has no doubt that the prompt use of large doses of *Morphia* saved his patient."

"S. L." translates a case of insanity related by Dr. H. Obersteiner, which ultimately recovered. His improvement was largely due to tobacco smoking, as claimed by the translator.

Dr. J. A. Hoffman writes upon diphtheria insisting strongly upon treating cases according to the totality of the symptoms and giving the remedy time to act. He denounces washes and gargles. Declares his opinion that many false cases of diphtheria are reported cured in the journals, especially tonsillitis, which is magnified into "malignant Diphtheria" with a big D. His indications for *Proto-iodide of Mercury* are: bilious temperament, moist tongue, enlarged tonsils, yellow false membrane, exudation thick and stringy with or without bronchial complications. For *Arsenicum* the symptoms are: putrefactive or typhoid form, glairy, red, shriveled and retracted tongue, throat dark, fœtid exudation, putrid and bloody discharge from the nose and ears, very tired and exhausted, tendency to collapse, or to a comatose condition.

Dr. Q. O. Sutherland reports a case of puerperal convulsions not relieved until after delivery of a dead, eight months' child by instruments, when they slowly disappeared.

*The British Journal of Homœopathy*, Jan., 1881,

Opens with an article upon dilutions in which the writer succeeds in filling much space with the dreary details of the ludicrous report upon the "Milwaukee Test." This is a marring of some otherwise interesting matter. Speaking of some odd changes of opinion frankly acknowledged by Dr. Skinner, the author gets off this following wise piece of philosophy: "There really seems something in the so-called 'Hahnemannism' which makes men arrogant and virulent beyond all measure; and few of its votaries escape the contagion"! Here the learned author of "Dilutions" mistakes the enthusiasm, impetuousness and perfect frankness of Dr. Skinner for arrogance and virulence. And even if the charge were true, he takes this one case as a basis for *generalizing* in reference to *all* the "Hahnemannians." He thus commits the same mistake that he makes in teaching homœopathy—that of *generalizing;* and hence the pages of the *British Journal* teem with empiricism only equalled by the dominant school that it so weakly imitates.

The address of Dr. Hayward at Liverpool is also given in this number. It deals with the "somewhat general and neutral subject of Fashions in Therapeutics."

Dr. Blake writes quite a learned article on "Rheumatic Gout and its Congeners," and teaches alternation of medicines. At the end of the article he actually advises the use of a quack remedy "of unknown constitution probably very complex in character, perhaps containing *Colchicum*"*!!* Perhaps we should not speak of this as we thereby expose the fact that we are tainted with the "contagion" of arrogance and virulence!

Dr. E. M. Hale reports curing a case of subacute myelitis with *Strychnine* in doses of one thirtieth of a grain, with the advice of an old school physician. The doctor claims it as homœopathic according to the pathological observations in Allen.

Dr. Dudgeon writes an article upon the temperature of the breath showing that when the bulb of a thermometer is wrapped up in cotton, silk or wool, and then breathed upon, the mercury rises to an unusual height. This phenomenon is found to depend upon the dryness of the fabric in consequence of which water is condensed and heat produced.

*The Medical Advance*, January, 1881.

Dr. John Hall of Toronto, reports the following cases:

Diphtheria in a little girl. Lachesis C.M. cured.

Scarlatina complicated with scarlet rash. Lachesis C.M. cured.

Intermittent fever. *Undefined development of chill and heat or both together.* Ars. 50 M. cured.

Intermittent fever. An exceedingly interesting case. Nat-mur. and then Gels., though indicated, failed. The following symptoms then appeared. *Pricking in anus duriug the fever and when sitting. Ascarides. Peevish. Pale yellowish face. Whitish clay-colored stools. Cold damp feet. Stomach swollen outwardly like an inverted saucer. Craving for eggs.* Calc-carb· 21 M. Much better. The case was finished with Gels. C.M.

Membranous dysmenorrhœa. The patient is much depressed in damp or wet weather. Very sensitive to cold damp air and changes in the weather. Cured with Rhus tox. 30 M. and 75 M.

Catarrh of the head with painful menstruation. Greatly relieved by Sil. 20 M., followed by Nux v.

Fistula in ano cured with Thuja 50 M., 80 M. and 5 M. then Silicea 20 M. and 40 M. A very interesting case to Hahnemannians.

Tabes mesenterica wonderfully improved by a single dose of Psorinum M.M. (Swan).

Dr. T. M. Watson reports a case of acute insanity brilliantly cured with Bell., Hyos., Lach. and Psorinum given on strict homœopathic principles.

Prof. J. R. Kippax relates a case of fracture and dislocation of nasal bone successfully replaced. Scirrhus of breast removed by the knife. Lupus erythematodes cured. Cervical endometritis cured with mechanical treatment (dilatation of cervix) and internal remedies. Indications not given.

A polycistic ovarian tumor cured without operation in eighteen months by homœopathic remedies—Apis mel., 6th. and 12th., followed by Lyc. 12th. and 30th.

Dr. Edward Rushmore gives six cases of intermittent fever cured respectively by Ars., Nux v., Puls., Eupatorium and Nat-mur., all given on strictly homœopathic principles.

G. N. Brigham cured two cases of uterine polypus with Thuja 2 C.

Dr. Blackburn relates two curious cases of negroes; one with white spots, itching and burning, relieved by Ars. 30—after giving the medicine black spots appearing upon the white ones—and the other, chills every month, accompanied by swelling of scrotum.

Dr. J. F. Edgar cured a case of ingrowing toe-nail with Marum Verum 4th., using no mechanical treatment whatever. In a few well chosen sentences he shows that this deformity depends upon pre-existing dynamic condition. His statements and conclusions are strictly correct.

Dr. Wilson had a case of apparent chronic suppurative inflammation of the middle ear with parasites in the meatus. Syringing with hot water to kill the parasites, and the internal administration of *Sulphur* produced a cure.

Dr. Grabill reports seven cases of intermittent fever cured with Opium, Cina, Lach., Nat-mur., Phos., Bell., Nux v. He denounces the use of Quinine with great vigor.

Dr. Nichols cured two cases of wen — one on the scalp the other on the upper eye-lid with Graphites C.M. and 2 c.

Dr. Davis writes upon senile hypertrophy of the prostate. He says that the treatment "must be palliative not curative." How does he know? By the tenor of his remarks he has never tried pure homœopathy, but contents himself with an assertion, borrowed from Gross. "Homœopathy is so young, and there are so few true practioners of its principles, that the incurability of this or any other disease is by no means certain." On the contrary, the remarkable achievements at the hands of the few who actually try the system, are calculated to make one dare attempt any feat in therapeutics. None of these diseases will ever be cured if we simply sit down and take for granted what old school doctors say about it.

*The Medico-Chirurgical Quarterly*, Jan., 1881.

Dr. Butler describes his invention for performing massage and giving induced currents of electricity at same time. Its use however, is a mere repetition of old school empiricism. He gives no symptoms by which we may be guided as to when it *is* indicated and when not.

Dr. Geo. S. Norton writes upon the use of boracic acid in chronic suppuration of the middle ear. He applies it locally in the powdered form while *at the same time* he gives Calc-phos. Sil., Hepar, Puls., etc. How can he tell *which* cured, the internal remedy or the externally applied boracic acid? Why does he not give one remedy at a time?

Two cases of asthma caused by the presence of a cat, are given at p. 147.

Dr. Kershaw cured a severe case of sciatica—*the least movement causing exqusite torture*—with Bryonia.

The editor also gives *The Organon* a severe castigation. He informs the profession that this journal sets up for its standard, "My doxy is orthodoxy and every other man's doxy is heterodoxy," and that it can not long survive. We hereby learn that the only true doxy in this world where reigns "liberty of medical opinion and action," is that which least represents one's measures. It is correct doxy to use a name which, to the vulgar mind, conveys some special, but hazy, idea of improved treatment, and to a professional mind some special *plan* of treatment, whilst the practioner at the same time, does as he pleases; that is, practices the empiricism that has been so signal a failure for a couple of thousand years. It is correct "doxy" to *assume* to practice a principle that by the laws of logic requires that you shall not use a drug for curative purposes until you have first found out something about its character and its sphere of action. That you shall give only one such remedial agent at a time, lest by giving more it leave a doubt in your mind as to *which* cured, and thus prevent your making a rapid cure in the next case you meet. It is correct "doxy" to *pretend* to follow a principle that contains within itself such requirements, and then in total disregard thereof, give "Woolridge's Tincture, a patent medicine of unknown constitution, probably very complex in character, perhaps containing colchicum."* It is good "doxy" to do this, even though the patient might have done it for himself, without the assistance of the learned champion of "Liberty of medical opinion and action." This is *not* "heterodoxy," it ain't. If you dare to call it so you are not giving "every one a chance to be heard." You are not advocating the "broadest principles of liberalism." You are not giving to others the "untrammeled liberty of thought, word and action." You are not carrying on "such discussion" in a "gentlemanly, courteous spirit."

*The Medical Counselor*, January, 1881.

Dr. Arnold reports a case of advanced pulmonary consumption cured by the administration Ars. alb., 3d., 6th. and 12th.

Dr. Heath shows how treatment of a case of convulsions in an old woman from overloaded stomach did not come under the operation of the law of similars. No, you must clean out the organ by causing vomiting with tartar emetic in three grain doses.

Dr. Boyle cured a case of diarrhœa of two weeks' standing by Petrol. 2 c.

Dr. House reports a case of apparent cholera in a farmer—rice water discharges, etc. It is suggested that the patient caught it whilst ministering to his own hogs which were sick of the cholera. Cured by Verat-alb. followed by Camphor, then Bell.,

* Vide *British Journal of Homœopathy*, Jan., 1881, p. 58.

and later, Nux v. The symptoms at first called for Verat. which was duly given. We think the doctor should have waited a little longer before giving the Camphoı. However, the patient was treated homœopathically, and the succession of the indicated remedies is a matter of judgment. This same physician also gives a case of skin disease cured with Ars. 2 c., and a case of gonorrhœal discharge re-established after a cold, cured by Medorrhine C.M. (Fincke).

*Hahnemannian Monthly*, January, 1881.

Dr. Laird reports a very interesting case of pulmonary abscess seemingly occurring as a consequence of mal-treatment of several successively appearing carbuncles. "The history of the case, the long series of aborted carbuncles followed by internal suppuration, the nocturnal paroxysms of dyspnœa with their peculiar concomitant symptoms, the prostration and hectic and the marked aggravation of all her sufferings after sleep, pointed unerringly to the remedy." Lachesis 41 M. Cured in about ten weeks.

Dr. Houghton reports two serious' cases of carries of temporal bone. From one of these a discharge of pus had continued for twenty-three years. They were successfully treated with a variety of remedies and by operative means.

Dr. Bell reports a case of fracture of femur in a child twenty-two months old.

Dr. Dake proposes to eradicate the germs of small-pox from a room where patients have been confined with it, by increase of heat and moisture so as to start up a fermentation which shall destroy the life of the germ.

Dr. Weiner reports provings made upon himself of Eucalyptus glob.

Dr. Scott writes an article entitled, "Is Shock an Element of Acute Disease?" He offers the propability of shock as an explanation of the sudden death occurring in the course of acute disease.

Dr. Bell reports his seventh case of ovariatomy which was treated after the operation, with Arnica, and then Verat. Result, complete recovery.

*The American Homœopath*, January, 1881.

Dr. Armstrong reports a case of valvular disease of the heart, and points out the value of observing more closely the pulsations in the jugular vein as showing tricuspid failure. His prescription of Digitalis 2 x is not accompanied by the proper symptomatic reasons, hence there is nothing to be learned from it.

Rev. Dr. Viehle gives an empirical prescription for frost bites. Thus doth homœopathy, like a crab, backward go.

Dr. Blake writes upon diarrhœa and gives a number of indications for remedies.

Dr. Pennoyer gives a case of vaginismus with delusion that her husband was going to murder her. Cured with Lachesis c.c.

Dr. Millie Chapman writes upon Stomatitis Materna, with cases. She depends upon feeding for cure, remedies being rather secondary.

